# Inverse Association between Air Pressure and Rheumatoid Arthritis Synovitis

**DOI:** 10.1371/journal.pone.0085376

**Published:** 2014-01-15

**Authors:** Chikashi Terao, Motomu Hashimoto, Moritoshi Furu, Shuichiro Nakabo, Koichiro Ohmura, Ran Nakashima, Yoshitaka Imura, Naoichiro Yukawa, Hajime Yoshifuji, Fumihiko Matsuda, Hiromu Ito, Takao Fujii, Tsuneyo Mimori

**Affiliations:** 1 Center for Genomic Medicine, Kyoto University Graduate School of Medicine, Kyoto, Japan; 2 Department of the Control for Rheumatic Diseases, Kyoto University Graduate School of Medicine, Kyoto, Japan; 3 Department of Rheumatology and Clinical Immunology, Kyoto University Graduate School of Medicine, Kyoto, Japan; Keio University School of Medicine, Japan

## Abstract

Rheumatoid arthritis (RA) is a bone destructive autoimmune disease. Many patients with RA recognize fluctuations of their joint synovitis according to changes of air pressure, but the correlations between them have never been addressed in large-scale association studies. To address this point we recruited large-scale assessments of RA activity in a Japanese population, and performed an association analysis. Here, a total of 23,064 assessments of RA activity from 2,131 patients were obtained from the KURAMA (Kyoto University Rheumatoid Arthritis Management Alliance) database. Detailed correlations between air pressure and joint swelling or tenderness were analyzed separately for each of the 326 patients with more than 20 assessments to regulate intra-patient correlations. Association studies were also performed for seven consecutive days to identify the strongest correlations. Standardized multiple linear regression analysis was performed to evaluate independent influences from other meteorological factors. As a result, components of composite measures for RA disease activity revealed suggestive negative associations with air pressure. The 326 patients displayed significant negative mean correlations between air pressure and swellings or the sum of swellings and tenderness (p = 0.00068 and 0.00011, respectively). Among the seven consecutive days, the most significant mean negative correlations were observed for air pressure three days before evaluations of RA synovitis (p = 1.7×10^−7^, 0.00027, and 8.3×10^−8^, for swellings, tenderness and the sum of them, respectively). Standardized multiple linear regression analysis revealed these associations were independent from humidity and temperature. Our findings suggest that air pressure is inversely associated with synovitis in patients with RA.

## Introduction

Rheumatoid arthritis (RA) is an autoimmune disorder characterized by joint synovitis and resultant joint destruction. Patients with RA present with swellings and tenderness of their joints, especially small joints such as metacarpophalangeal joints and proximal interphalangeal joints. Joint swellings and tenderness are closely related with future joint destruction [1], [2] and this is why joint swellings and tenderness are included in the items for composite measures used for evaluation of RA activity [3]–[6]. Disease activity index (DAS) 28 is the most common composite measure in RA used for evaluation of daily RA activity and is composed of erythrocyte sedimentation rate (ESR) or C-reactive protein (CRP) as inflammatory parameters, swollen joint count (SJC) and tender joint count (TJC) for the 28 joints with or without visual analogue scale (VAS).

A large number of studies have tried to elucidate basic mechanisms of joint synovitis in RA and revealed the importance of inflammatory cytokines such as TNF-alpha and IL-6 to which biological agents were developed to target [7]. On the contrary, environmental factors which influence joint synovitis are scarcely known. Several studies with a relatively large number of subjects have reported seasonal variations of RA symptoms or joint destruction [8], [9], but the relationship is still inconclusive [10]–[12]. Detailed meteorological differences among various seasons which cause changes in RA symptoms have not been clarified.

Through our daily medical care for patients with RA, we noticed that many patients with RA told us about the fluctuations in their joint symptoms according to changes in air pressure. In particular, many of them recognized worsening of their symptoms when the air pressure decreased such as during a typhoon in the summer. While many rheumatologists have heard of this, there have been no large-scale association studies to date addressing the relationship between air pressure and joint synovitis in RA. Previous studies addressing correlations between RA synovitis and meteorological changes included less than 100 patients with RA and the results were not consistent [10], [13]–[18]. Previously, it was reported that a consistent microenvironment would ease joint symptoms in patients with RA [13]. While one study showed that temperature and humidity were associated with joint pain in 88 patients with RA [15], another study did not find statistically significant associations between meteorological changes and arthritic symptoms in 70 patients [16]. Another study where researchers observed 18 patients with RA for more than one year did not find significant associations and the authors concluded that this subjective belief in association by RA patients is simply an assumption that people have believed in for thousands of years [19]. However, none of the studies analyzed a large-number of joint assessments and the association is still inconclusive.

Recently, Kyoto University developed a large-scale database named “KURAMA (Kyoto University Rheumatoid Arthritis Management Alliance)” to accumulate detailed clinical information and specimen of patients with RA to uncover the basics of RA [20]. Here, we obtained 23,064 joint assessments for patients with RA from the KURAMA database and analyzed correlations between air pressure and joint synovitis in RA.

## Results

Firstly, whether air pressure was correlated with daily disease activity in RA was addressed. DAS28 was selected as evaluation of RA activity for the association study. Table 1 shows the basic characteristics of the DAS28 scores and its components. The mean DAS28 score in the current patient group was 3.28, indicating low to moderate disease activity [21]. 14,999 DAS28 scores with three variables including SJC, TJC and ESR did not show a significant association with air pressure (p = 0.18, Table S1). Because DAS is a composite measure for assessing disease activity in RA, other RA composite measures could be associated with air pressure. Correlation analyses between air pressure and SJC, TJC, ESR, patients' VAS (pVAS) and Dr's VAS (dVAS) showed that all elements except for ESR showed suggestive or significant inverse associations with air pressure (Table S1). We also found that the sum of SJC and TJC showed a significant association with air pressure (Table S1). These results suggest that air pressure is associated with RA synovitis across different evaluations and different patients with RA. However, these analyses might be influenced by intra-patient correlations. Considering that significant associations were observed for SJC and the sum of SJC and TJC with air pressure, and that the largest number of data was available for SJC and TJC among the components of composite measures, we adopted SJC, TJC, and the sum of the two counts as best candidates showing associations with meteorological factors to obtain the maximum power for further analyses.

**Table 1:** Basic characteristics of the subjects in the current study.

Items	Overall	326 patients
Evaluation	23,064	12,061
Patient	2,131	326
Age (mean±SD)	60.7±15.0	62.4±13.6
Female ratio (%)	80.9	82.5
Stage* (mean±SD)	2.63±1.18	2.86±1.16
Class* (mean±SD)	1.92±0.70	1.94±0.70
Disease duration (year, mean±SD)	14.0±11.9	16.1±11.7
Smoking** (%)	33.6	30.8
Biologics*** (%)	19.0	46.6

Steinbrocker's Stage and Class.

Current smoker and ex-smoker.

Patients who have been treated by biological agents between 2005 and 2012.

SD:standard deviation.

**Figure 1:**
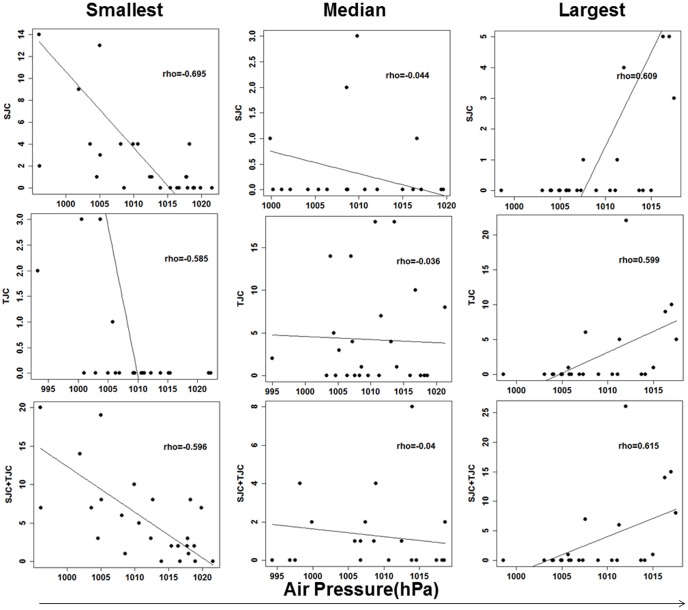
Correlations between joint synovitis and air pressure in RA patients. Correlation plots in patients demonstrating the smallest, median and largest correlation coefficients between joint synovitis and air pressure are illustrated in the left, middle and right panels, respectively.

Secondly, correlation between air pressure and RA synovitis in each patient was analyzed to control intra-patient correlations. As the distribution of number of evaluations varied in the patients with RA, we extracted patients with more than 20 evaluations to confirm the correlations between air pressure and joint synovitis across different patients. In total, 12,061 evaluations from 326 patients were used for this analysis. The means of SJC and TJC were comparable with those in the 2,131 patients (Table 1). The overall fluctuations between SJC, TJC, or the combination of the two and air pressure are illustrated in Figure S1. Because we could not find the regular strong tendency of association between air pressure and joint synovitis through the figure, correlation coefficients between air pressure and signs of joint synovitis were calculated for each of the 326 patients. The correlation coefficients in items of synovitis demonstrated normal distributions in each item (p≥0.78, Shapiro-Wilk test, data not shown), justifying the application of t-test. The mean correlation coefficients across the 326 patients revealed significant negative correlations of air pressure with SJC and the combination of SJC with TJC (mean rho = −0.0410 and −0.0455, p = 0.00068 and 0.00011, respectively, Table 2). TJC showed a suggestive negative correlation (mean rho = −0.0306 and p = 0.010, Table 2). Figure 1 illustrates the smallest, largest and median correlation coefficients between air pressure and SJC, TJC or combination of the two among the 326 patients with RA, suggesting that negative mean correlation coefficients of RA synovitis were not brought about by patients demonstrating extreme negative correlation coefficients.

**Table 2:** Mean correlation coefficients between joint synovitis and air pressure in the 326 patients.

Synovitis	Mean±SD(median)	Mean ρ±SD	P*
SJC	2.07±1.99 (1)	−0.0410±0.210	0.00068
TJC	2.08±2.19 (1)	−0.0306±0.212	0.010
SJC+TJC	4.15±3.70 (2)	−0.0455±0.207	0.00011

p-values for Student's t-test. SD:standard deviation.

**Figure 2:**
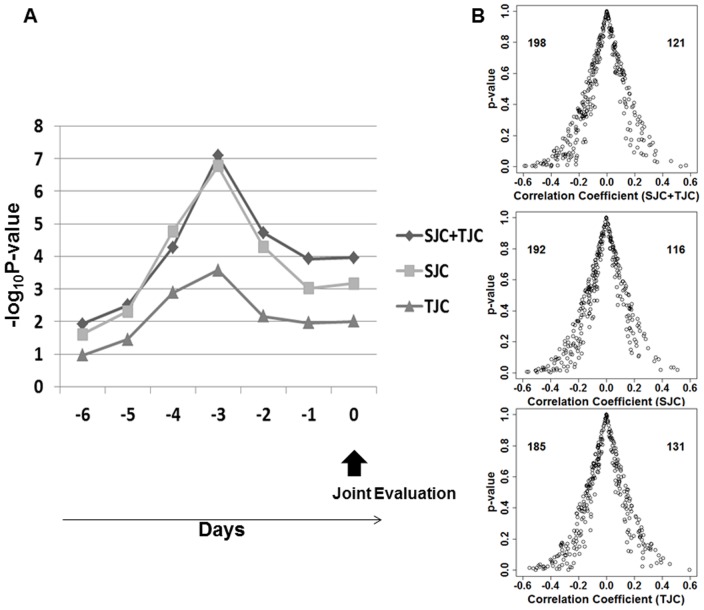
The strongest associations between joint synovitis and air pressure three days before joint evaluations. The 326 patients with more than 20 evaluations were analyzed. A)Associations between air pressure and joint synovitis for the seven consecutive days. Y axis indicates −log_10_P-value of Student's t-test. X axis indicates days before joint evaluations. B)Volcano plots for distribution of RA patients demonstrating positive or negative associations between joint synovitis and air pressure three days before joint evaluations. X and Y axes indicate Spearman's correlation coefficient and P-value, respectively. Numbers in the panels indicate RA patients showing positive or negative correlation coefficients. Because a part of the patients showed consistent TJC or SJC across all evaluations, the sum of the two numbers in each panel does not make 326.

Thirdly, we analyzed which day showed the most significant associations between air pressure and items of joint synovitis, because it was likely that air pressure affected RA joint synovitis by an indirect mechanism taking several days. The air pressure data of six consecutive days before the date of evaluation was obtained and the same analyses using the same data set of the 326 patients were performed to evaluate associations with items of joint synovitis. As a result, the mean correlation coefficients of SJC, TJC, and the sum of SJC and TJC showed a “U-pattern” in the consecutive days (Figure 2A). The strongest associations were found three days before the joint evaluations for the three items (p = 1.7×10^−7^, 0.00027, and 8.3×10^−8^, for SJC, TJC, and the sum of SJC and TJC, respectively, Figure 2A and 2B). These results strengthened correlations between air pressure and joint synovitis in RA.

Finally, whether these correlations were mainly brought about by other meteorological factors or not was addressed. Data of daily mean temperatures and humidity were obtained from the same period as air pressure. Multiple standardized linear regression analyses were performed to assess independent correlations between joint synovitis and air pressure. As a result, air pressure showed significant associations with joint synovitis in the 21,940 evaluations which were independent from temperature and humidity (beta≤−0.0765 and p≤0.0031, Table 3). The analyses suggested that humidity also negatively influenced RA joint synovitis (Table 3). When multiple standardized linear regression analyses were performed for air pressure three days before the evaluations in each of the 326 patients with more than 20 evaluations of the 28 joints, mean coefficients of air pressure showed significant negative associations with joint synovitis (p = 0.00023, 0.036 and 0.0015, for SJC, TJC and the sum of SJC and TJC, respectively, Table 4). The inverse association between humidity and air pressure was also observed in this analysis (p = 0.0019, 0.016 and 0.0023, for SJC, TJC and the sum of SJC and TJC, respectively, data not shown).

**Table 3:** Results of multiple standardized linear regression analysis between air pressure and joint synvoitis.

Synovitis	-	Air pressure (hPa)		Temperature (°C)		Humidity (%)	
	Mean±SD(median)	1009.34±6.42 (1009.25)		16.10±8.77 (16.45)		63.83±9.75 (64)	
	-	Beta	P	Beta	P	Beta	P
SJC	1.96±3.06 (1)	−0.0765	0.0031	−0.0419	0.097	−0.0996	2.8×10^−6^
TJC	2.12±3.70 (1)	−0.105	0.00082	−0.0535	0.079	−0.0976	0.00015
SJC+TJC	4.08±5.87 (2)	−0.181	0.00027	−0.0954	0.049	−0.197	1.4×10^−6^

**Table 4:** Standardized multiple linear regression analysis for air pressure 3 days before evaluations and synovitis among the 326 patients.

Synovitis	Mean±SD(median)	mean Beta±SD	P*
SJC	2.07±1.99 (1)	−0.126±0.0425	0.00023
TJC	2.08±2.19 (1)	−0.0893±0.0339	0.036
SJC+TJC	4.15±3.70 (2)	−0.215±0.0672	0.0015

p-values for Student's t-test. SD:standard deviation.

## Discussion

Because environmental effects on RA synovitis are not well established, analysis with convincing results would be beneficial to manage patients with RA properly. This is the first study to address the correlations between air pressure and joint synovitis in RA with a large number of RA patients, and the first to detect significant associations between them. Our results indicate that low air pressure is associated with worsening of joint synovitis. This matches the complaints from RA patients, that they feel worsening of RA synovitis when typhoons come in the summer. Since SJC is an objective element and TJC is a subjective element of patients with RA, significant associations of air pressure with the two cannot be explained by subjective feelings of patients. Previous studies addressing correlations between meteorological effects and joint synovitis did not give consistent results [10], [13]–[19]. The limited number of subjects (not more than 88 patients with RA) is assumed to have caused this inconsistency. In addition, although we found significant associations, the small mean correlation coefficients (ρ: −0.04∼) made the previous studies difficult to detect these small effects. Because RA patients at Kyoto University Hospital frequently told us of the fluctuations in their symptoms in accordance with air pressure, among all the various meteorological factors we focused on air pressure. Another reason we only focused on air pressure was to avoid type I statistical error by performing multiple association studies.

Our data set revealed low to moderate disease activity of RA on average, which reflects the appropriateness of the treatment the patients were receiving at Kyoto Unvesity Hospital. Considering large variation of the synovitis data and large number of joint evaluations, the current data should be enough to detect correlations.

The first analysis addressing correlation between air pressure and DAS28 did not result in a significant association. However, it should be noted that all of the elements of composite measures showed the same negative direction of association with air pressure. These results may suggest common associations between air pressure and RA joint synovitis instead of limited association between air pressure and a specific element of RA joint synovitis. At the same time, these results may suggest that composite measures composed of multiple elements showing weak association with air pressure are not sensitive enough to detect the influence of air pressure. In addition, because many medical doctors evaluate synovitis of many patients with RA, variations among doctors in each element would be amplified in the composite measure and result in difficulty to detect significant correlations. Considering the strong association between air pressure and sum of SJC and TJC, the fine direction of association between different elements and air pressure should partly overlap but partly differ. PVAS showed the lowest mean correlation coefficient among items of composite measures. This matches our observation that many of the RA patients feel correlation between their symptoms and air pressure. Although the number of data for pVAS is limited, 81 patients with more than 15 data for pVAS revealed a suggestive inverse correlation with air pressure (mean rho = −0.0469, data not shown). Accumulation of more data for each element would detect a significant association between each element and air pressure and a fine direction of association with air pressure in each element.

Because the number of joint assessments differed among patients and it was likely that analyses using all assessments would be influenced by the specific patients with large number of joint assessments, we performed the analyses focusing on the 326 patients with more than 20 assessments to avoid intra-patient correlations. We hypothesized that correlations between air pressure and joint synovitis should be largely different among patients but a large number of patients would result in a significant deviation. We calculated a correlation coefficient in each patient separately. The results supported our hypothesis and showed a significant deviation of the mean correlation coefficient from the null hypothesis. Figure 1 presenting patients with median correlations along with Table S2 indicated that overall distributions of correlations between air pressure and joint synovitis shifted to negative correlations. Considering the size of correlation coefficients, although RA patients show a negative correlation in average between air pressure and joint synovitis, the correlation greatly vary among patients and the correlation should not be generalized.

We found the strongest associations between air pressure and signs of synovitis three days before evaluations. This may indicate that slow mechanisms underlie the correlations or that joint synovitis is prone to unknown factors which reflect past air pressure.

When we classified patients into subgroups based on positivity of disease duration, smoking, Stage, Class and usage of biological agents during the observation period, we did not find significant difference among RA subsets (data not shown). The analysis of confounding factors revealed that the association of air pressure with joint synovitis was not derived from humidity and temperature, which were selected since air pressure, humidity and temperature are representatives of meteorological factors. Although both humidity and temperature showed correlations with air pressure (rho:0.19 and 0.58, between air pressure and humidity or temperature, respectively), their correlations could not explain the association between air pressure and RA synovitis. The analysis also revealed that humidity showed a negative association with joint synovitis that is independent from temperature and air pressure. A previous study reported that a combination of increase in humidity and decrease in air pressure were associated with worsening of joint pain [14]. Their findings matched our results for air pressure, but the association of humidity was opposite to ours. Thus, the association between humidity and joint synovitis is inconclusive and further studies are required. It is notable that mean temperature was not associated with joint synovitis in a multiple standardized linear regression analysis. As increase or decrease of blood flow due to temperature is supposed to influence signs of synovitis in RA, the lack of the association may be explained by rapid influence of finely conditioned temperature in hospitals.

It is difficult to assume the basic mechanisms underlying the correlation between joint synovitis and air pressure. One possible explanation is that air pressure directly presses joint structures in patients with RA. Low air pressure results in reduced outside pressure of joints which allow joints to be swollen more easily. Enlarged space of joints would allow more inflammatory cells to enter joint space and produce inflammatory cytokines. Another explanation is involvement of autonomic nerves to regulate threshold of pain. A Japanese group reported that both decreased air pressure and temperature led to worsening of joint pain in an animal model [22], [23]. The group also showed that these correlations in the animal model were mediated by sympathetic nerve, whose excitement and increase of circulating noradrenaline were brought about by decrease of air pressure [24], [25]. As the current study did not reveal a strong association between joint tenderness and air pressure, involvement of sympathetic nerve with pain cannot fully explain the current results. Variations of B-cell activity due to meteorological changes could be another possibility. Our previous study reported seasonal variation of IgG in rheumatic diseases [26]. Analysis incorporating altitude of residence for each patient, whose information is not available in the current study, would give a clue for the mechanism underlying the association.

We could not conclude whether air pressure directly influences RA synovitis or if it is just a confounding factor of yet-to-be-determined factors with direct effects on RA synovitis. However, our analysis supports the patients' subjective feelings of relationship between air pressure and joint synovitis. Another study addressing correlations between air pressure and joint synvoitis estimated by imaging including ultrasound seems necessary. Further experiments and analyses between air pressure and joint symptoms in humans would clarify the detailed mechanisms. It will be interesting to determine the characteristics of patients who are susceptible to change of air pressure.

## Materials and Methods

### Ethical statements

The analyses in the current study were performed under policy of data analysis of the KURAMA database approved by Kyoto University Hospital Ethical Committee [20]. Written informed consent to enroll in the database described below was obtained from most of the patients, but for some patients the information regarding the construction of this database was disclosed instead of obtaining written informed consent. Participants who were informed regarding the construction of the database (instead of obtaining written informed consent) were allowed to withdraw from the study if desired. All data were de-identified and analyzed anonymously. This study was designed in accordance with the Helsinki Declaration.

### Data of joint synovotis in patients with RA

A total of 23,064 evaluations of disease activity from 2,131 patients with RA were obtained with the corresponding dates of evaluations from the KURAMA database. The evaluation data contained some or all of the SJC, TJC in the 28 joints, ESR, pVAS and dVAS as well as DAS 28 as a composite measure for RA disease activity. The sum of SJC and TJC was also calculated for each evaluation. 326 patients with more than 20 evaluations of disease activity were extracted for further analysis.

### Data of air pressure and other meteorological factors

Data of daily mean air pressure in Kyoto from 2005 to 2012 was obtained from the homepage of Japan Meteological Agency (http://www.jma.go.jp/jma/index.html). Data of daily mean temperature and humidity in Kyoto were also obtained in the same manner.

### Statistical analysis

Correlations between mean air pressure on the day of joint evaluation and DAS28, SJC, TJC, ESR, pVAS, dVAS or sum of SJC and TJC were estimated by Spearman's correlation coefficients, using 23,064 evaluations of joint synovitis or evaluations in each of the 326 patients. The mean Spearman's correlation coefficients among the 326 patients with RA were estimated by Student's t-test under the null hypothesis that the mean was zero. Normality of distribution of correlation coefficients in the 326 patients was analyzed by Shapiro-Wilk test. A daily mean air pressure of the six days before the day of joint evaluation was also analyzed for correlations with signs of joint synovitis across the 326 patients with RA. To analyze independent effects of air pressure on joint synovitis from humidity and temperature, multiple standardized linear regression analysis was performed for 23,064 evaluations and each of the 326 patients with more than 20 evaluations. Mean beta values in the multiple standardized linear regression analysis among the 326 patients were assessed by Student's t-test under the null hypothsis that the mean beta values were zero. P-values less than 0.0071 were regarded as significant based on Bonferroni's correction. Data analysis was performed by R software (http://www.r-project.org/) or SPSS (ver 18).

## Supporting Information

Figure S1 **Fluctuations of air pressure and joint synovitis in the 326 patients.** Fluctuations of each item are illustrated between 2005 and 2012. The three figures of SJC, TJC and combination of SJC and TJC are composed of 326 lines presenting fluctuations in the 326 patients.
(TIF)

Table S1 **Correlation coefficients of RA joint synovitis in association with air pressure across different evaluations.**
(DOC)

Table S2 **Detailed information of the 326 RA patients.**
(DOC)
