# Supplementary figures and images for: Inverse Association between Air Pressure and Rheumatoid Arthritis Synovitis
Source: PLoS One. 2014 Jan 15;9(1):e85376. doi: 10.1371/journal.pone.0085376 (PMC3893195; doi:10.1371/journal.pone.0085376)

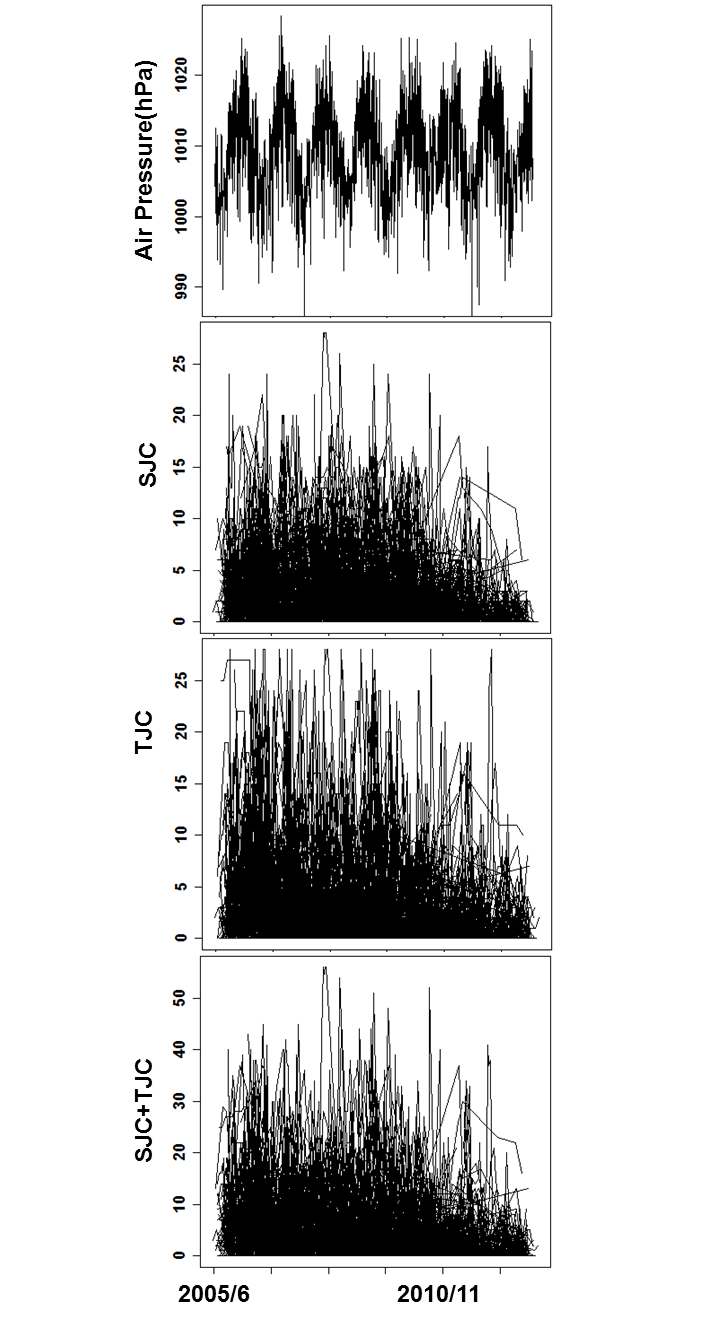

Supplement: Figure S1 — Fluctuations of air pressure and joint synovitis in the 326 patients. Fluctuations of each item are illustrated between 2005 and 2012. The three figures of SJC, TJC and combination of SJC and TJC are composed of 326 lines presenting fluctuations in the 326 patients. (TIF) [file pone.0085376.s001.tif]
