# Supplementary material for: Inverse Association between Air Pressure and Rheumatoid Arthritis Synovitis
Source: PLoS One. 2014 Jan 15;9(1):e85376. doi: 10.1371/journal.pone.0085376 (PMC3893195; doi:10.1371/journal.pone.0085376)
Supplement: Table S1 — Correlation coefficients of RA joint synovitis in association with air pressure across different evaluations. (DOC) [file pone.0085376.s002.doc]

| Item | Number | Mean SD(median) | ρ | P** |
| --- | --- | --- | --- | --- |
| Air Pressure | - | 1009.34±6.42 (1009.25) | - | - |
| DAS28* | 14999 | 3.28±1.25 (3.16) | -0.0101 | 0.18 |
| SJC | 21940 | 1.96±3.06 (1) | -0.0152 | 0.024 |
| TJC | 21940 | 2.12±3.70 (1) | -0.0191 | 0.0047 |
| SJC+TJC | 21940 | 4.08±5.87 (2) | -0.0207 | 0.0022 |
| pVAS | 6973 | 34.98±25.95 (30) | -0.0351 | 0.0034 |
| dVAS | 7114 | 17.65±18.23 (11) | -0.0220 | 0.064 |
| ESR | 15835 | 28.03±25.69 (18) | -0.0035 | 0.66 |

*DAS28 using three variables, namely, SJC, TJC and ESR

**p-value of Spearman’s correlation coefficient
