# Supplementary material for: Inverse Association between Air Pressure and Rheumatoid Arthritis Synovitis
Source: PLoS One. 2014 Jan 15;9(1):e85376. doi: 10.1371/journal.pone.0085376 (PMC3893195; doi:10.1371/journal.pone.0085376)
Supplement: Table S2 — Detailed information of the 326 RA patients. (DOC) [file pone.0085376.s003.doc]

| Patient | Evaluation* | SJC** | CorrSJC*** | TJC** | CorrTJC*** | SJC+TJC** | CorrSJC+TJC*** |
| --- | --- | --- | --- | --- | --- | --- | --- |
| pt1 | 24 | 0±0 (0) | NA | 0.13±0.34 (0) | 0.009 | 0.13±0.34 (0) | 0.009 |
| pt2 | 23 | 1±2.17 (0) | -0.646 | 1.87±3.31 (0) | -0.553 | 2.87±5.36 (0) | -0.556 |
| pt3 | 36 | 0.03±0.17 (0) | 0.102 | 0.03±0.17 (0) | -0.238 | 0.06±0.24 (0) | -0.098 |
| pt4 | 23 | 0.83±1.67 (0) | 0.609 | 2.57±5.23 (0) | 0.599 | 3.39±6.69 (0) | 0.615 |
| pt5 | 23 | 1.87±1.66 (2) | 0.039 | 0.74±1.05 (0) | 0.202 | 2.61±2.29 (2) | 0.104 |
| pt6 | 36 | 0.92±1.2 (1) | 0.096 | 1.08±1.27 (1) | 0.022 | 2±2.32 (1) | 0.099 |
| pt7 | 32 | 0.31±0.59 (0) | 0.015 | 0.72±0.96 (0) | -0.082 | 1.03±1.23 (0.5) | -0.077 |
| pt8 | 28 | 0.46±0.96 (0) | -0.446 | 1.07±1.39 (0.5) | 0.038 | 1.54±1.86 (1) | -0.226 |
| pt9 | 48 | 5.67±3.17 (6) | -0.342 | 4.75±3.08 (4) | -0.105 | 10.42±5.79 (11) | -0.264 |
| pt10 | 49 | 1.08±1.3 (1) | -0.034 | 3.86±5.61 (2) | 0.14 | 4.94±5.78 (3) | 0.122 |
| pt11 | 82 | 2.32±4.12 (2) | 0.178 | 0.9±3.3 (0) | 0.026 | 3.22±6.36 (2) | 0.158 |
| pt12 | 43 | 1.51±1.26 (1) | -0.1 | 4.3±4.22 (3) | -0.056 | 5.81±4.82 (5) | -0.059 |
| pt13 | 27 | 1.4±1.5 (1) | 0.305 | 0.96±1.79 (0) | 0.342 | 2.36±2.87 (1) | 0.356 |
| pt14 | 45 | 1.8±0.87 (2) | -0.097 | 1±0.88 (1) | -0.189 | 2.8±1.29 (2) | -0.204 |
| pt15 | 20 | 0.2±0.62 (0) | -0.231 | 1.1±1.94 (0) | 0.001 | 1.3±2.03 (0.5) | -0.04 |
| pt16 | 28 | 1.81±1.52 (2) | 0.32 | 0.37±1.39 (0) | 0.143 | 2.19±2.22 (2) | 0.328 |
| pt17 | 33 | 1.64±0.96 (2) | -0.061 | 1.58±1.39 (1) | -0.195 | 3.21±2.1 (3) | -0.167 |
| pt18 | 23 | 3.57±3.4 (3) | -0.373 | 7.61±5.18 (7) | -0.286 | 11.17±7.93 (9) | -0.335 |
| pt19 | 20 | 1.9±3.95 (0) | -0.183 | 2.3±5.38 (0) | -0.295 | 4.2±9.29 (0) | -0.2 |
| pt20 | 23 | 1.43±0.73 (1) | -0.055 | 0.91±0.67 (1) | 0.25 | 2.35±1.07 (2) | 0.06 |
| pt21 | 84 | 1.36±1.43 (1) | 0.006 | 1.31±2.13 (0) | 0.107 | 2.68±2.92 (2) | 0.065 |
| pt22 | 28 | 0.26±0.53 (0) | -0.082 | 1.33±2.08 (0) | -0.008 | 1.59±2.47 (0) | -0.022 |
| pt23 | 25 | 2.55±1.84 (2) | -0.065 | 1.77±1.8 (1) | 0.109 | 4.32±3.41 (3.5) | 0.009 |
| pt24 | 45 | 1.98±2.15 (1) | -0.135 | 2.33±2.41 (1) | -0.319 | 4.3±4.44 (2) | -0.295 |
| pt25 | 126 | 1.46±3.2 (0) | 0.029 | 2.29±4.24 (0) | -0.127 | 3.75±6.89 (0) | -0.096 |
| pt26 | 66 | 2.21±1.82 (2) | -0.106 | 1.17±1.24 (1) | -0.07 | 3.38±2.62 (3) | -0.131 |
| pt27 | 34 | 0.12±0.41 (0) | 0.266 | 2.5±3.89 (2) | -0.037 | 2.62±4.08 (2) | -0.012 |
| pt28 | 78 | 1.24±1.42 (1) | 0.007 | 0.97±1.36 (0) | 0.107 | 2.22±2.58 (2) | 0.051 |
| pt29 | 26 | 1.09±1.48 (0.5) | -0.196 | 2.09±1.82 (2) | -0.119 | 3.18±2.84 (3) | -0.174 |
| pt30 | 20 | 0.35±0.81 (0) | -0.044 | 0.85±1.27 (0) | -0.005 | 1.2±1.79 (0) | -0.025 |
| pt31 | 62 | 1.05±1.08 (1) | -0.004 | 0.39±0.78 (0) | 0.065 | 1.44±1.55 (1) | 0.019 |
| pt32 | 41 | 1.12±0.98 (1) | -0.257 | 1.2±1.47 (1) | -0.093 | 2.32±1.98 (2) | -0.254 |
| pt33 | 23 | 0.13±0.63 (0) | 0.257 | 0.13±0.63 (0) | 0.257 | 0.26±1.25 (0) | 0.257 |
| pt34 | 25 | 2.4±3.33 (2) | 0.235 | 6.12±6.02 (4) | -0.06 | 8.52±7.67 (6) | 0.006 |
| pt35 | 31 | 1.65±2.18 (1) | 0.118 | 0.29±0.74 (0) | -0.142 | 1.94±2.43 (1) | 0.054 |
| pt36 | 23 | 0.91±1 (1) | 0.453 | 0.57±0.99 (0) | 0.241 | 1.48±1.62 (1) | 0.402 |
| pt37 | 37 | 0.65±0.72 (1) | 0.168 | 0.38±0.98 (0) | -0.003 | 1.03±1.34 (1) | 0.16 |
| pt38 | 39 | 0.67±0.87 (0) | 0.2 | 0.67±1.03 (0) | 0.144 | 1.33±1.75 (0) | 0.184 |
| pt39 | 20 | 0.95±0.22 (1) | 0.338 | 0.2±0.41 (0) | -0.412 | 1.15±0.49 (1) | -0.224 |
| pt40 | 53 | 0.89±1.65 (0) | -0.104 | 1.42±2.6 (0) | -0.085 | 2.3±4.11 (0) | -0.077 |
| pt41 | 64 | 1.8±1.75 (1) | -0.254 | 3.05±2.12 (3) | -0.155 | 4.84±3.36 (4) | -0.215 |
| pt42 | 20 | 0.55±1.82 (0) | 0.242 | 0.55±1.28 (0) | 0.301 | 1.1±3.02 (0) | 0.309 |
| pt43 | 38 | 0.82±0.69 (1) | -0.044 | 1.63±1.17 (1) | 0.133 | 2.45±1.59 (2) | 0.094 |
| pt44 | 69 | 1.45±1.49 (1) | -0.05 | 2±1.96 (2) | -0.053 | 3.45±3.19 (3) | -0.046 |
| pt45 | 81 | 4.79±4.01 (5) | 0.083 | 2.95±3.73 (2) | 0.001 | 7.74±6.99 (6) | 0.072 |
| pt46 | 51 | 0.94±1.1 (1) | 0.156 | 2.16±2.13 (2) | 0.087 | 3.1±2.71 (2) | 0.146 |
| pt47 | 40 | 1±0.93 (1) | -0.164 | 1.4±0.81 (2) | 0.079 | 2.4±1.3 (2) | -0.086 |
| pt48 | 29 | 0±0 (0) | NA | 0.52±0.78 (0) | -0.404 | 0.52±0.78 (0) | -0.404 |
| pt49 | 43 | 3.72±2.58 (4) | 0.012 | 2.16±2.75 (1) | 0.109 | 5.88±4.24 (5) | 0.077 |
| pt50 | 40 | 1.68±1.9 (1) | 0.147 | 1.45±2.71 (1) | 0.008 | 3.13±4.18 (2) | 0.073 |
| pt51 | 22 | 0.18±0.39 (0) | -0.111 | 0.95±2.28 (0) | 0.099 | 1.14±2.38 (0) | 0.036 |
| pt52 | 40 | 0.03±0.16 (0) | 0.257 | 0.28±0.72 (0) | 0.166 | 0.3±0.79 (0) | 0.17 |
| pt53 | 64 | 7.14±4.4 (7) | -0.212 | 6.7±5.07 (6) | -0.151 | 13.84±8.89 (13) | -0.188 |
| pt54 | 55 | 1.07±1.53 (0) | 0.104 | 0.84±1.3 (0) | 0.046 | 1.91±2.44 (1) | 0.078 |
| pt55 | 77 | 2.61±2.2 (2) | -0.185 | 2.74±2.62 (2) | -0.19 | 5.35±4.54 (4) | -0.226 |
| pt56 | 26 | 0.15±0.46 (0) | -0.101 | 0.12±0.43 (0) | 0.013 | 0.27±0.67 (0) | -0.124 |
| pt57 | 32 | 2.66±1.1 (2) | 0.181 | 1.56±1.52 (1) | 0.025 | 4.22±2.12 (4) | 0.092 |
| pt58 | 35 | 2.46±3.59 (1) | -0.036 | 2.49±4.06 (0) | -0.05 | 4.94±7.4 (2) | -0.04 |
| pt59 | 38 | 0.16±0.44 (0) | -0.043 | 0.32±0.7 (0) | 0.036 | 0.47±0.92 (0) | 0.067 |
| pt60 | 105 | 4.52±3.6 (4) | -0.184 | 2.17±3.28 (1) | -0.048 | 6.7±6.14 (6) | -0.157 |
| pt61 | 25 | 0.44±1.19 (0) | -0.169 | 1.16±1.62 (0) | -0.337 | 1.6±2.57 (0) | -0.328 |
| pt62 | 24 | 0.38±0.77 (0) | 0.02 | 0.67±1.4 (0) | 0.333 | 1.04±1.85 (0) | 0.195 |
| pt63 | 32 | 0.63±1.16 (0) | -0.299 | 1.06±1.92 (0) | -0.299 | 1.69±2.92 (0) | -0.293 |
| pt64 | 25 | 5.76±5.3 (4) | -0.149 | 7.12±5.49 (6) | -0.288 | 12.88±9.28 (10) | -0.259 |
| pt65 | 73 | 1.63±1.3 (1) | 0.053 | 1.84±1.36 (2) | -0.07 | 3.47±1.66 (3) | -0.046 |
| pt66 | 52 | 1.02±2.73 (0) | 0.271 | 1.92±3.03 (1) | 0.116 | 2.94±5.45 (1) | 0.174 |
| pt67 | 48 | 3.15±2.64 (2.5) | 0 | 3.58±3.26 (2) | 0.16 | 6.73±5.56 (4.5) | 0.091 |
| pt68 | 20 | 0.35±0.59 (0) | -0.185 | 0.5±0.83 (0) | -0.23 | 0.85±1.23 (0) | -0.156 |
| pt69 | 20 | 5.05±1.54 (5.5) | -0.163 | 4.4±2.48 (4.5) | 0.064 | 9.45±3.69 (9.5) | -0.014 |
| pt70 | 20 | 0.65±0.49 (1) | 0.373 | 0.4±0.5 (0) | -0.053 | 1.05±0.89 (1) | 0.166 |
| pt71 | 20 | 0.25±0.55 (0) | 0.115 | 1.5±1.76 (1) | -0.431 | 1.75±2.1 (1) | -0.382 |
| pt72 | 62 | 2.45±2.38 (2) | 0.108 | 2.33±3.05 (1) | 0.106 | 4.78±4.97 (3) | 0.111 |
| pt73 | 86 | 1.12±1.28 (1) | 0.178 | 2.28±1.88 (2) | -0.064 | 3.4±2.6 (3) | 0.093 |
| pt74 | 23 | 4.48±5.42 (1) | 0.214 | 8.09±8.7 (6) | 0.515 | 12.57±13.21 (12) | 0.476 |
| pt75 | 25 | 3.52±3.25 (2) | -0.137 | 3.16±2.81 (2) | -0.334 | 6.68±5.77 (4) | -0.199 |
| pt76 | 44 | 0.36±0.61 (0) | -0.175 | 1.89±1.94 (2) | 0.396 | 2.25±2.21 (2) | 0.282 |
| pt77 | 52 | 1.52±2.65 (0) | -0.236 | 5.25±3.08 (5) | -0.296 | 6.77±5.15 (5) | -0.351 |
| pt78 | 22 | 0.59±1.62 (0) | 0.316 | 1.45±1.5 (1.5) | -0.319 | 2.05±2.36 (2) | -0.124 |
| pt79 | 22 | 1.59±1.22 (2) | 0.025 | 0.05±0.21 (0) | 0.327 | 1.64±1.29 (2) | 0.039 |
| pt80 | 42 | 0.21±0.47 (0) | -0.234 | 0.29±0.71 (0) | 0.079 | 0.5±0.86 (0) | -0.102 |
| pt81 | 24 | 1.08±1.38 (0) | 0.104 | 1.29±2.12 (0) | 0.121 | 2.38±3.05 (1.5) | 0.108 |
| pt82 | 79 | 1.18±1.03 (1) | -0.102 | 0.99±0.78 (1) | 0.199 | 2.17±1.55 (2) | 0.032 |
| pt83 | 73 | 7.04±7.05 (5) | -0.039 | 8.77±7.35 (7) | 0.021 | 15.81±13.77 (11) | -0.002 |
| pt84 | 58 | 1.9±1.82 (2) | 0.293 | 0.98±1.66 (0) | 0.319 | 2.88±3.13 (2) | 0.327 |
| pt85 | 34 | 1.76±1.76 (1) | 0.036 | 2.12±2.24 (1.5) | -0.281 | 3.88±3.62 (2.5) | -0.21 |
| pt86 | 40 | 0.55±1.04 (0) | -0.175 | 0.5±0.72 (0) | 0.016 | 1.05±1.45 (0) | -0.091 |
| pt87 | 33 | 2.7±1.94 (3) | -0.248 | 2.06±1.56 (2) | 0.05 | 4.76±2.74 (5) | -0.095 |
| pt88 | 72 | 3.07±3.34 (2) | 0.221 | 3.25±4.97 (1) | 0.108 | 6.32±6.88 (4) | 0.17 |
| pt89 | 33 | 0.36±0.7 (0) | 0.088 | 0.67±0.99 (0) | -0.107 | 1.03±1.38 (0) | -0.032 |
| pt90 | 45 | 0.31±0.56 (0) | -0.292 | 0.22±0.47 (0) | -0.35 | 0.53±0.99 (0) | -0.307 |
| pt91 | 27 | 0.44±0.97 (0) | -0.127 | 0.15±0.46 (0) | 0.127 | 0.59±1.01 (0) | -0.081 |
| pt92 | 30 | 0.1±0.4 (0) | 0.186 | 0.5±0.97 (0) | 0.227 | 0.6±1.19 (0) | 0.236 |
| pt93 | 33 | 0.73±0.88 (0) | -0.251 | 0.36±0.55 (0) | 0.109 | 1.09±1.1 (1) | -0.163 |
| pt94 | 21 | 1.29±2.08 (0) | -0.006 | 1.24±2.7 (0) | -0.191 | 2.52±4.63 (1) | -0.142 |
| pt95 | 98 | 7.15±4.62 (6) | 0.083 | 6.57±5.19 (6) | 0.189 | 13.72±7.29 (13) | 0.22 |
| pt96 | 67 | 2.13±2.19 (2) | 0.117 | 1.82±2.28 (1) | -0.133 | 3.96±3.91 (3) | -0.009 |
| pt97 | 45 | 3.18±4.82 (2) | -0.242 | 7.22±8.05 (4) | 0.013 | 10.4±11.65 (6) | -0.063 |
| pt98 | 35 | 0.89±1.13 (0) | 0.076 | 4±5.88 (1) | -0.137 | 4.89±6.52 (2) | -0.083 |
| pt99 | 27 | 0.19±0.48 (0) | -0.259 | 0.22±0.58 (0) | -0.257 | 0.41±1.05 (0) | -0.255 |
| pt100 | 33 | 1.94±1.73 (2) | -0.349 | 0.77±0.99 (0) | -0.272 | 2.71±2.21 (2) | -0.356 |
| pt101 | 28 | 1±1.78 (0) | 0.106 | 2.25±2.61 (1.5) | -0.139 | 3.25±4.1 (2) | -0.055 |
| pt102 | 56 | 1.57±2.21 (0) | -0.174 | 0.57±1.19 (0) | -0.048 | 2.14±3.15 (0) | -0.168 |
| pt103 | 72 | 1.83±3.03 (0) | -0.013 | 1.38±2.42 (0) | -0.013 | 3.21±5.1 (1) | 0.018 |
| pt104 | 24 | 1.39±1.47 (1) | -0.095 | 1.78±2.02 (1) | -0.037 | 3.17±3.23 (1) | -0.022 |
| pt105 | 24 | 0.83±1.13 (0) | -0.514 | 0.79±1.32 (0) | -0.252 | 1.63±2.26 (1) | -0.456 |
| pt106 | 46 | 0.41±0.65 (0) | -0.302 | 0.83±1.08 (0) | -0.11 | 1.24±1.37 (1) | -0.233 |
| pt107 | 29 | 1.14±1.36 (1) | -0.089 | 2.38±4.13 (1) | -0.243 | 3.52±5.11 (2) | -0.192 |
| pt108 | 73 | 3.85±2.77 (4) | 0.015 | 2.49±1.97 (2) | -0.077 | 6.34±4.14 (6) | -0.045 |
| pt109 | 30 | 3.03±3.64 (1.5) | 0.136 | 3.6±3 (3) | 0.263 | 6.63±6.02 (4.5) | 0.271 |
| pt110 | 71 | 1.01±1.84 (0) | 0.125 | 5.1±7.02 (1) | 0.085 | 6.11±7.4 (3) | 0.12 |
| pt111 | 28 | 1.18±1.16 (1) | -0.24 | 1.75±1.32 (2) | -0.09 | 2.93±1.72 (3) | -0.201 |
| pt112 | 68 | 2.62±2.95 (1.5) | 0.077 | 0.88±2.02 (0) | -0.052 | 3.5±4.31 (2) | 0.067 |
| pt113 | 39 | 1.54±1.98 (1) | -0.032 | 0.9±1.23 (0) | 0.214 | 2.44±2.73 (2) | 0.085 |
| pt114 | 36 | 0.26±0.66 (0) | 0.008 | 3.34±4.15 (2) | -0.009 | 3.6±4.25 (2) | -0.008 |
| pt115 | 30 | 1.66±2.04 (1) | -0.119 | 3±3.36 (2) | -0.415 | 4.66±4.84 (4) | -0.39 |
| pt116 | 39 | 0.67±1.44 (0) | -0.424 | 0.85±1.8 (0) | -0.487 | 1.51±3.16 (0) | -0.491 |
| pt117 | 30 | 0.3±0.6 (0) | -0.121 | 0.1±0.31 (0) | -0.045 | 0.4±0.77 (0) | -0.116 |
| pt118 | 21 | 0.57±1.66 (0) | -0.25 | 0.67±2.06 (0) | -0.25 | 1.24±3.71 (0) | -0.25 |
| pt119 | 37 | 3.89±2.91 (4) | 0.152 | 1.03±1.48 (0) | 0.162 | 4.92±3.89 (4) | 0.152 |
| pt120 | 75 | 2.11±2.6 (1) | -0.011 | 5.25±3.61 (5) | 0.071 | 7.37±5.76 (6) | 0.043 |
| pt121 | 24 | 2.96±3.38 (2) | -0.401 | 4.54±4.33 (3) | 0.16 | 7.5±7.12 (5) | -0.19 |
| pt122 | 59 | 2.18±1.6 (2) | -0.024 | 0.71±0.74 (1) | -0.047 | 2.89±1.82 (3) | -0.012 |
| pt123 | 85 | 5.15±3.39 (5) | -0.121 | 4.49±2.3 (4) | -0.076 | 9.64±4.89 (9) | -0.114 |
| pt124 | 45 | 4.16±3.67 (4) | 0.162 | 0.8±1.31 (0) | 0.22 | 4.96±4.44 (5) | 0.174 |
| pt125 | 45 | 3.8±2.75 (4) | -0.067 | 0.98±1.51 (0) | 0.304 | 4.78±3.52 (5) | 0.038 |
| pt126 | 29 | 0.24±0.44 (0) | -0.058 | 0.48±0.74 (0) | -0.568 | 0.72±0.96 (0) | -0.436 |
| pt127 | 34 | 1.56±1.21 (1) | 0.132 | 1.65±1.35 (1) | -0.142 | 3.21±2.24 (3) | -0.089 |
| pt128 | 83 | 1.7±0.64 (2) | -0.051 | 1.59±0.95 (2) | -0.131 | 3.29±1.3 (4) | -0.138 |
| pt129 | 71 | 1.47±1.33 (2) | 0.181 | 0.43±0.78 (0) | 0.086 | 1.9±1.8 (2) | 0.199 |
| pt130 | 91 | 1.74±2.04 (1) | 0.024 | 1.23±2.24 (0) | -0.147 | 2.97±3.83 (2) | -0.07 |
| pt131 | 45 | 0.98±1.37 (0) | 0.309 | 0.89±1.23 (0) | 0.27 | 1.87±2.31 (1) | 0.329 |
| pt132 | 36 | 1.08±1.2 (1) | 0.011 | 1.61±1.59 (2) | -0.142 | 2.69±2.49 (2) | -0.095 |
| pt133 | 25 | 0.92±0.91 (1) | -0.084 | 0.72±0.84 (0) | 0.288 | 1.64±1.41 (2) | 0.104 |
| pt134 | 26 | 3.75±2.52 (3) | -0.205 | 0.58±1.25 (0) | 0.018 | 4.33±3.51 (4) | -0.245 |
| pt135 | 48 | 4.36±4.76 (2.5) | 0.349 | 8.3±7.46 (8) | 0.292 | 12.66±11.22 (10.5) | 0.381 |
| pt136 | 24 | 2.38±2.16 (2) | -0.164 | 1.33±2.01 (0.5) | -0.079 | 3.71±3.87 (2) | -0.176 |
| pt137 | 20 | 2.3±1.42 (2) | -0.066 | 1.4±1.76 (1) | 0.042 | 3.7±2.79 (2.5) | -0.054 |
| pt138 | 65 | 2.43±1.99 (2) | -0.065 | 2.05±1.6 (2) | 0.08 | 4.48±2.8 (4) | 0.02 |
| pt139 | 26 | 4.35±3.22 (4) | -0.097 | 2.5±2.64 (2) | -0.25 | 6.85±5.39 (6) | -0.191 |
| pt140 | 40 | 0.51±0.84 (0) | 0.125 | 0.81±1.27 (0) | -0.04 | 1.32±1.86 (0) | 0.003 |
| pt141 | 101 | 1.91±1.73 (2) | 0.119 | 1.04±1.33 (1) | 0.001 | 2.95±2.86 (2) | 0.089 |
| pt142 | 20 | 1.25±1.68 (0) | 0.04 | 0.5±1.61 (0) | -0.324 | 1.75±2.75 (0) | -0.021 |
| pt143 | 51 | 0.88±1.11 (1) | -0.144 | 0.94±0.76 (1) | -0.035 | 1.82±1.53 (1) | -0.144 |
| pt144 | 21 | 0.62±0.67 (1) | 0.232 | 0.62±0.74 (0) | -0.281 | 1.24±1.18 (1) | -0.012 |
| pt145 | 60 | 1.35±3 (0) | -0.347 | 2.45±4.07 (0) | -0.352 | 3.8±6.81 (0) | -0.355 |
| pt146 | 86 | 0.68±1.09 (0) | 0.058 | 0.91±1.11 (0) | 0.075 | 1.59±1.69 (1) | 0.045 |
| pt147 | 35 | 0.86±1.61 (0) | 0.019 | 0.77±2.1 (0) | 0.069 | 1.63±3.63 (0) | 0.031 |
| pt148 | 28 | 1.61±1.23 (2) | 0.003 | 1.46±0.79 (2) | 0.024 | 3.07±1.9 (4) | 0.023 |
| pt149 | 40 | 0±0 (0) | NA | 0.4±1.66 (0) | -0.041 | 0.4±1.66 (0) | -0.041 |
| pt150 | 42 | 0.9±1.3 (0) | 0.321 | 0.62±0.82 (0) | -0.065 | 1.52±1.85 (1) | 0.175 |
| pt151 | 51 | 0.53±0.99 (0) | -0.051 | 0.84±1.59 (0) | 0.076 | 1.37±2.12 (0) | -0.021 |
| pt152 | 27 | 1.11±1.31 (0) | 0.001 | 2.81±3.78 (2) | 0 | 3.93±4.19 (4) | 0.158 |
| pt153 | 39 | 0.11±0.4 (0) | -0.192 | 0±0 (0) | NA | 0.11±0.4 (0) | -0.192 |
| pt154 | 50 | 3.8±3.87 (3) | -0.048 | 4.66±4.2 (4) | -0.062 | 8.45±7.92 (7) | -0.073 |
| pt155 | 36 | 3.28±2.89 (3) | -0.223 | 1±1.79 (0) | -0.202 | 4.28±3.65 (4) | -0.295 |
| pt156 | 33 | 0.45±0.71 (0) | 0.089 | 2.7±3.38 (2) | -0.249 | 3.15±3.44 (2) | -0.26 |
| pt157 | 37 | 1.11±1.37 (1) | -0.258 | 1.24±1.21 (1) | -0.089 | 2.35±2.07 (2) | -0.229 |
| pt158 | 54 | 2.56±2.05 (2) | -0.084 | 2.83±3.26 (2) | 0.047 | 5.39±5 (4) | 0.011 |
| pt159 | 28 | 0.71±2.32 (0) | 0.072 | 0.68±2.13 (0) | 0.158 | 1.39±4.25 (0) | 0.132 |
| pt160 | 61 | 1.44±1.71 (1) | -0.146 | 3.68±3.29 (3) | -0.124 | 5.12±4.1 (4) | -0.181 |
| pt161 | 22 | 3.77±2.52 (3.5) | -0.436 | 1.09±1.11 (1) | 0.205 | 4.86±3.04 (4.5) | -0.33 |
| pt162 | 44 | 0.64±0.99 (0) | -0.262 | 4.59±4 (4) | 0.031 | 5.23±4.32 (5) | -0.022 |
| pt163 | 54 | 5.39±5.42 (3) | -0.168 | 4.94±5.13 (4) | -0.209 | 10.33±9.57 (8) | -0.198 |
| pt164 | 79 | 3.9±2.1 (4) | -0.291 | 1.93±1.98 (1) | -0.106 | 5.84±3.73 (5) | -0.228 |
| pt165 | 50 | 5.56±2.89 (5) | -0.442 | 5.36±3.9 (5) | -0.285 | 10.92±6.27 (10) | -0.392 |
| pt166 | 26 | 7.54±4.73 (8.5) | 0.245 | 5.15±4.99 (4) | -0.089 | 12.69±8.54 (13) | 0.107 |
| pt167 | 45 | 2.6±1.44 (3) | -0.143 | 1.22±1.33 (1) | 0.077 | 3.82±2.14 (4) | -0.079 |
| pt168 | 22 | 0.45±2.13 (0) | -0.293 | 3.86±5.04 (2) | 0.247 | 4.32±5.12 (3) | 0.073 |
| pt169 | 28 | 3.57±2.95 (3) | 0.154 | 1.5±2.27 (0) | 0.369 | 5.07±4.86 (4) | 0.204 |
| pt170 | 26 | 6.05±2.74 (5.5) | -0.185 | 3.85±3.57 (3) | 0.15 | 9.9±5.71 (8) | -0.023 |
| pt171 | 31 | 1.7±1.71 (1) | -0.431 | 1.44±1.22 (1) | -0.255 | 3.15±2.49 (2) | -0.46 |
| pt172 | 28 | 1±0.48 (1) | -0.242 | 0.63±0.56 (1) | -0.041 | 1.63±0.74 (2) | -0.264 |
| pt173 | 25 | 0.96±1.08 (0.5) | 0.041 | 2.04±2.51 (1) | -0.196 | 3±2.54 (2) | 0.018 |
| pt174 | 35 | 4.21±2.37 (5) | -0.132 | 3.9±3.74 (3) | 0.256 | 8.1±5.56 (8) | 0.112 |
| pt175 | 26 | 2.6±3.07 (2) | 0.42 | 1.4±1.83 (1) | 0.102 | 4±4.39 (2) | 0.32 |
| pt176 | 22 | 4.77±5.29 (2.5) | -0.361 | 3±4.83 (1) | -0.005 | 7.77±7.88 (5) | -0.34 |
| pt177 | 22 | 1.82±2.24 (1) | -0.122 | 1.5±0.96 (1) | -0.397 | 3.32±2.28 (3) | -0.352 |
| pt178 | 25 | 3.92±4.48 (2) | -0.078 | 9.16±9.97 (0) | -0.151 | 13.08±13.83 (5) | -0.091 |
| pt179 | 24 | 7.8±5.69 (6) | -0.154 | 4.75±6.24 (2) | -0.171 | 12.55±11.52 (9) | -0.206 |
| pt180 | 35 | 0.38±0.82 (0) | 0.318 | 0.34±0.67 (0) | -0.164 | 0.72±1.1 (0) | 0.107 |
| pt181 | 28 | 0.19±0.48 (0) | -0.089 | 1±1.57 (0) | -0.165 | 1.19±1.84 (0) | -0.176 |
| pt182 | 22 | 2.32±1.29 (2) | -0.485 | 1.18±1.14 (1) | -0.463 | 3.5±2.09 (3.5) | -0.528 |
| pt183 | 36 | 1.74±2.45 (0) | -0.131 | 1.48±2.45 (0) | -0.328 | 3.23±4.43 (1) | -0.235 |
| pt184 | 23 | 0±0 (0) | NA | 0±0 (0) | NA | 0±0 (0) | NA |
| pt185 | 21 | 0.33±0.73 (0) | 0.008 | 2.14±4.08 (0) | -0.354 | 2.48±4.56 (1) | -0.312 |
| pt186 | 42 | 0.88±1.9 (0) | -0.258 | 3.48±4.33 (2) | -0.063 | 4.36±5 (2) | -0.145 |
| pt187 | 23 | 5.57±4.96 (5) | 0.466 | 0.48±1.08 (0) | 0.413 | 6.04±5.56 (6) | 0.499 |
| pt188 | 27 | 1.5±1.14 (1.5) | -0.07 | 1.73±1.19 (2) | -0.111 | 3.23±1.95 (3) | -0.073 |
| pt189 | 24 | 3.58±2.32 (3.5) | -0.24 | 3.17±1.76 (3) | 0.101 | 6.75±3.57 (6) | -0.118 |
| pt190 | 27 | 1.92±2.9 (0.5) | 0.257 | 1.58±3.3 (0) | 0.307 | 3.5±5.92 (1.5) | 0.234 |
| pt191 | 28 | 0±0 (0) | NA | 0.12±0.43 (0) | 0.09 | 0.12±0.43 (0) | 0.09 |
| pt192 | 20 | 4.8±1.7 (5) | -0.028 | 3.3±1.69 (3) | -0.423 | 8.1±3.11 (7.5) | -0.139 |
| pt193 | 32 | 0±0 (0) | NA | 0.09±0.39 (0) | -0.162 | 0.09±0.39 (0) | -0.162 |
| pt194 | 27 | 1.56±2.62 (1) | -0.113 | 9.88±4.91 (10) | -0.13 | 11.44±5.61 (12) | -0.208 |
| pt195 | 36 | 4.17±3.69 (2) | -0.054 | 4.39±4.15 (4) | -0.064 | 8.56±7.2 (6) | -0.092 |
| pt196 | 37 | 2.11±1.49 (2) | -0.13 | 1.08±1.16 (1) | -0.291 | 3.19±2.01 (3) | -0.255 |
| pt197 | 34 | 0.15±0.61 (0) | -0.276 | 0.21±0.88 (0) | -0.279 | 0.35±1.41 (0) | -0.322 |
| pt198 | 20 | 1.75±3.09 (0) | -0.079 | 2.15±3.51 (0) | -0.061 | 3.9±6.54 (0) | -0.058 |
| pt199 | 40 | 6.23±3.02 (7) | -0.041 | 3.74±2.98 (3) | 0.117 | 9.97±5.26 (9) | 0.084 |
| pt200 | 23 | 2.25±4.91 (0) | 0.168 | 2.65±5.32 (0) | 0.025 | 4.9±9.83 (0) | 0.068 |
| pt201 | 37 | 8.11±5.24 (9) | 0.017 | 1.38±2.11 (0) | 0.071 | 9.49±6.2 (10) | -0.033 |
| pt202 | 44 | 2.02±1.62 (2) | 0.189 | 0.83±1.28 (0) | 0.183 | 2.85±2.26 (2) | 0.303 |
| pt203 | 20 | 0±0 (0) | NA | 0.65±1.84 (0) | -0.329 | 0.65±1.84 (0) | -0.329 |
| pt204 | 23 | 0.78±1.24 (0) | -0.099 | 0.09±0.29 (0) | 0.012 | 0.87±1.39 (0) | -0.109 |
| pt205 | 26 | 2.44±2.2 (1) | 0.073 | 2.32±1.75 (2) | -0.047 | 4.76±3.72 (3) | 0.051 |
| pt206 | 38 | 3.92±1.67 (4) | 0.067 | 1.66±1.48 (1) | -0.022 | 5.58±2.26 (5) | 0.036 |
| pt207 | 36 | 4.22±4.96 (2) | -0.032 | 1.06±1.41 (0) | -0.176 | 5.28±5.59 (3.5) | -0.138 |
| pt208 | 42 | 2.83±1.58 (3) | -0.192 | 1.33±1.68 (1) | -0.064 | 4.17±2.69 (4) | -0.177 |
| pt209 | 32 | 0.09±0.3 (0) | -0.296 | 0.09±0.3 (0) | -0.168 | 0.19±0.47 (0) | -0.258 |
| pt210 | 41 | 1.41±1.88 (0) | -0.3 | 1.8±2.15 (1) | 0.075 | 3.22±3.55 (3) | -0.134 |
| pt211 | 20 | 0.8±1.11 (0) | -0.043 | 1.8±1.85 (1.5) | -0.469 | 2.6±2.48 (2) | -0.335 |
| pt212 | 26 | 4.8±3.03 (5) | 0.021 | 1.68±1.77 (1) | -0.06 | 6.48±4.28 (5) | 0.008 |
| pt213 | 20 | 0.65±0.93 (0) | -0.373 | 2.8±2.61 (2) | 0.208 | 3.45±2.61 (3) | 0.121 |
| pt214 | 22 | 0±0 (0) | NA | 0±0 (0) | NA | 0±0 (0) | NA |
| pt215 | 26 | 5±2.43 (5) | 0.052 | 4.67±3.47 (3) | 0.175 | 9.67±5.49 (8) | 0.129 |
| pt216 | 35 | 0.14±0.36 (0) | -0.146 | 1.11±2.87 (0) | 0.052 | 1.26±2.86 (0) | -0.006 |
| pt217 | 31 | 2.63±3.45 (2) | 0.021 | 0.44±0.75 (0) | 0.254 | 3.07±3.69 (3) | 0.047 |
| pt218 | 43 | 2.49±4.39 (1) | -0.118 | 1.3±2.88 (0) | -0.098 | 3.79±6.52 (2) | -0.123 |
| pt219 | 20 | 0.25±0.72 (0) | 0.121 | 1.15±1.04 (1) | 0.005 | 1.4±1.39 (1) | 0.04 |
| pt220 | 25 | 2.62±2.64 (1) | -0.107 | 3.57±3.38 (2) | -0.41 | 6.19±5.69 (5) | -0.365 |
| pt221 | 22 | 0.2±0.52 (0) | -0.149 | 0.25±0.72 (0) | -0.011 | 0.45±1.19 (0) | -0.128 |
| pt222 | 21 | 0.25±0.55 (0) | 0.147 | 0.05±0.22 (0) | -0.179 | 0.3±0.57 (0) | 0.049 |
| pt223 | 55 | 2.52±2.42 (2) | -0.082 | 3.81±5.65 (1.5) | 0.031 | 6.33±7.35 (3) | -0.033 |
| pt224 | 21 | 1.05±1.16 (1) | -0.016 | 2.57±2.54 (2) | -0.231 | 3.62±3.56 (2) | -0.155 |
| pt225 | 34 | 1.91±1.23 (2) | 0.062 | 0.67±0.89 (0) | -0.259 | 2.58±1.84 (2) | -0.117 |
| pt226 | 23 | 5.35±3.42 (6) | -0.344 | 2.91±2.13 (3) | 0.013 | 8.26±4.79 (7) | -0.236 |
| pt227 | 20 | 0.15±0.49 (0) | -0.068 | 0.25±0.55 (0) | 0.042 | 0.4±0.94 (0) | 0.082 |
| pt228 | 42 | 2.9±2.14 (2.5) | 0.141 | 2.36±2.53 (2) | 0.044 | 5.26±4.29 (4) | 0.139 |
| pt229 | 42 | 2.47±1.43 (3) | -0.311 | 1.42±1.33 (1) | 0.004 | 3.89±2.3 (4) | -0.174 |
| pt230 | 22 | 3.86±4.12 (2) | -0.213 | 2±3.51 (1) | -0.48 | 5.86±7.36 (3.5) | -0.282 |
| pt231 | 34 | 2.94±2.04 (3) | -0.211 | 1.47±2.02 (1) | -0.218 | 4.41±3.73 (4) | -0.303 |
| pt232 | 24 | 5.08±2.57 (4) | 0.023 | 2.83±1.76 (2.5) | -0.377 | 7.92±3.82 (6) | -0.153 |
| pt233 | 34 | 0±0 (0) | NA | 0.68±1.53 (0) | 0.248 | 0.68±1.53 (0) | 0.248 |
| pt234 | 34 | 0.97±1.65 (0) | 0.053 | 1.21±1.45 (1) | 0.059 | 2.18±2.76 (1) | 0.038 |
| pt235 | 22 | 1.15±0.99 (1) | -0.005 | 0.45±1 (0) | 0.499 | 1.6±1.5 (2) | 0.301 |
| pt236 | 42 | 0.02±0.15 (0) | 0.148 | 5.86±4.79 (4) | -0.189 | 5.88±4.79 (4) | -0.185 |
| pt237 | 24 | 1.54±1.44 (2) | 0.096 | 0.08±0.41 (0) | -0.075 | 1.63±1.41 (2) | 0.063 |
| pt238 | 24 | 2.29±4.44 (0) | -0.462 | 0.17±0.64 (0) | -0.365 | 2.46±4.85 (0) | -0.468 |
| pt239 | 46 | 0.43±0.94 (0) | -0.374 | 0.36±0.53 (0) | -0.236 | 0.79±1.34 (0) | -0.295 |
| pt240 | 25 | 0.12±0.33 (0) | 0.017 | 1.56±3.99 (0) | 0 | 1.68±4.01 (0) | -0.031 |
| pt241 | 68 | 0.06±0.36 (0) | 0.069 | 0.02±0.13 (0) | 0.093 | 0.08±0.45 (0) | 0.07 |
| pt242 | 32 | 5.83±2.16 (6) | 0.384 | 1.45±1.7 (1) | 0.113 | 7.28±3.18 (7) | 0.284 |
| pt243 | 66 | 2.25±3.78 (0) | 0.054 | 1.85±3.37 (0) | 0.089 | 4.1±6.79 (1) | 0.042 |
| pt244 | 31 | 0.2±0.41 (0) | 0.462 | 0.43±1.3 (0) | 0.383 | 0.63±1.5 (0) | 0.533 |
| pt245 | 28 | 4.04±2.66 (4) | -0.28 | 1.57±1.57 (1) | -0.084 | 5.61±3.53 (6) | -0.227 |
| pt246 | 20 | 0.1±0.31 (0) | -0.087 | 0.4±0.82 (0) | 0.123 | 0.5±1 (0) | 0.113 |
| pt247 | 25 | 0.6±0.87 (0) | -0.37 | 0.24±0.44 (0) | -0.104 | 0.84±1.14 (0) | -0.329 |
| pt248 | 36 | 5.06±2.98 (5) | -0.291 | 0.46±1.24 (0) | -0.115 | 5.51±3.22 (5) | -0.345 |
| pt249 | 33 | 1.39±0.93 (1) | -0.29 | 0.7±0.77 (1) | 0.128 | 2.09±1.42 (2) | -0.116 |
| pt250 | 28 | 1.04±1.65 (0) | 0.041 | 4.3±3.69 (4) | 0.209 | 5.33±4.67 (4) | 0.189 |
| pt251 | 30 | 0±0 (0) | NA | 0±0 (0) | NA | 0±0 (0) | NA |
| pt252 | 25 | 0±0 (0) | NA | 0±0 (0) | NA | 0±0 (0) | NA |
| pt253 | 30 | 1.7±2.78 (0) | 0.06 | 2±3.41 (0) | 0.023 | 3.7±6.08 (0) | 0.05 |
| pt254 | 30 | 1.17±1.78 (0) | -0.175 | 2.47±4.79 (0.5) | -0.173 | 3.63±5.1 (2) | -0.223 |
| pt255 | 33 | 4.32±2.26 (4.5) | 0.34 | 6.61±3.84 (6) | 0.199 | 10.93±5.65 (10.5) | 0.295 |
| pt256 | 58 | 2.38±1.23 (3) | 0.094 | 1.29±1.3 (1) | -0.186 | 3.67±2.06 (3.5) | -0.04 |
| pt257 | 26 | 4.16±2.41 (4) | -0.458 | 3±2.12 (3) | -0.315 | 7.16±4.11 (7) | -0.478 |
| pt258 | 26 | 0.04±0.2 (0) | -0.027 | 4.23±5.87 (1) | -0.036 | 4.27±5.85 (1.5) | -0.035 |
| pt259 | 77 | 3.55±4.16 (2) | -0.228 | 0.47±0.97 (0) | -0.296 | 4.01±4.89 (2) | -0.27 |
| pt260 | 22 | 1.95±1.43 (2) | 0.248 | 0.24±0.62 (0) | -0.02 | 2.19±1.5 (2) | 0.243 |
| pt261 | 29 | 1.41±1.21 (1) | 0.272 | 1.17±0.8 (1) | 0.298 | 2.59±1.55 (3) | 0.366 |
| pt262 | 57 | 5.72±4.7 (4.5) | -0.184 | 6.57±4.21 (7.5) | -0.262 | 12.3±8.29 (12.5) | -0.254 |
| pt263 | 22 | 0±0 (0) | NA | 0±0 (0) | NA | 0±0 (0) | NA |
| pt264 | 26 | 4.1±4.99 (2) | 0.136 | 4.38±5.18 (2) | -0.114 | 8.48±10.01 (4) | -0.025 |
| pt265 | 47 | 8.63±5.7 (8) | 0.421 | 9.12±5.63 (8) | 0.375 | 17.76±10.74 (18) | 0.41 |
| pt266 | 26 | 0.12±0.59 (0) | 0.067 | 2.08±3.07 (0) | -0.198 | 2.19±3.16 (0) | -0.174 |
| pt267 | 22 | 1.5±1.5 (1.5) | -0.238 | 1.95±2.38 (2) | -0.001 | 3.45±3.32 (2) | -0.142 |
| pt268 | 25 | 0±0 (0) | NA | 0±0 (0) | NA | 0±0 (0) | NA |
| pt269 | 30 | 0.37±0.79 (0) | -0.493 | 0.19±0.56 (0) | -0.377 | 0.56±1.28 (0) | -0.498 |
| pt270 | 24 | 6.42±3.88 (6) | 0.131 | 6.71±7.15 (4) | 0.248 | 13.13±7.9 (11) | 0.275 |
| pt271 | 20 | 1.35±1.14 (1) | -0.241 | 1.35±0.81 (1) | -0.146 | 2.7±1.78 (2.5) | -0.211 |
| pt272 | 31 | 4.94±2.69 (4) | 0.115 | 3.39±2.8 (4) | -0.114 | 8.32±4.66 (8) | 0.051 |
| pt273 | 72 | 1.56±2.03 (1) | 0.226 | 12.97±11.05 (12.5) | -0.066 | 14.53±10.99 (19) | -0.032 |
| pt274 | 49 | 2.02±2.28 (1) | -0.062 | 0.72±1.19 (0) | -0.152 | 2.74±3.1 (1.5) | -0.088 |
| pt275 | 30 | 0.33±0.71 (0) | -0.033 | 0.23±0.68 (0) | 0.383 | 0.57±1.14 (0) | 0.083 |
| pt276 | 21 | 1.19±1.57 (0) | 0.023 | 1.43±1.69 (1) | 0.226 | 2.62±2.94 (2) | 0.148 |
| pt277 | 23 | 0.09±0.42 (0) | 0.225 | 0.65±1.37 (0) | 0.278 | 0.74±1.63 (0) | 0.292 |
| pt278 | 21 | 2.48±1.47 (2) | -0.321 | 1.76±1.67 (2) | -0.402 | 4.24±2.93 (4) | -0.46 |
| pt279 | 24 | 0.14±0.47 (0) | 0.319 | 1.32±1.32 (1) | -0.173 | 1.45±1.53 (1) | -0.106 |
| pt280 | 25 | 2.64±3.93 (1) | -0.695 | 2.88±2.33 (2) | -0.436 | 5.52±5.55 (3) | -0.596 |
| pt281 | 22 | 4.62±7.05 (0) | -0.113 | 5.14±7.95 (0) | -0.086 | 9.76±14.96 (0) | -0.126 |
| pt282 | 25 | 2.25±3.03 (1) | -0.171 | 1.71±2.01 (1) | 0.107 | 3.96±4.68 (2) | -0.065 |
| pt283 | 36 | 4.31±2.57 (4) | -0.043 | 0.86±1.1 (0) | -0.165 | 5.17±3.33 (5) | -0.092 |
| pt284 | 27 | 0.63±0.93 (0) | -0.211 | 1.59±1.5 (1) | 0.213 | 2.22±2.06 (1) | 0.034 |
| pt285 | 22 | 0±0 (0) | NA | 0±0 (0) | NA | 0±0 (0) | NA |
| pt286 | 26 | 1±1.41 (0) | 0.148 | 0±0 (0) | NA | 1±1.41 (0) | 0.148 |
| pt287 | 26 | 0.65±1.16 (0) | -0.172 | 0.19±0.57 (0) | -0.09 | 0.85±1.57 (0) | -0.177 |
| pt288 | 43 | 2.47±2.03 (2) | 0.02 | 1.79±2.25 (1) | -0.018 | 4.26±3.96 (3) | 0.012 |
| pt289 | 25 | 1.52±3.03 (1) | -0.189 | 1.28±2.37 (0) | -0.346 | 2.8±5.22 (1) | -0.234 |
| pt290 | 40 | 4.93±1.89 (4) | 0.057 | 4.5±2.65 (4) | 0.108 | 9.43±4.26 (8) | 0.089 |
| pt291 | 25 | 6.82±4.11 (6) | -0.179 | 3.41±5.18 (2) | -0.086 | 10.23±8.47 (8) | -0.086 |
| pt292 | 24 | 0.96±1.49 (0) | -0.01 | 1.83±2.37 (0) | 0.027 | 2.79±3.41 (1.5) | 0.035 |
| pt293 | 24 | 0.17±0.48 (0) | 0.341 | 1.67±1.99 (1) | 0.216 | 1.83±2.35 (1) | 0.214 |
| pt294 | 38 | 7.34±4.67 (6) | 0.085 | 5.84±4.36 (5) | -0.131 | 13.18±6.23 (11) | -0.098 |
| pt295 | 22 | 2.62±2.16 (2) | -0.11 | 0.43±0.98 (0) | -0.585 | 3.05±2.69 (2) | -0.314 |
| pt296 | 48 | 0.34±0.56 (0) | 0.076 | 0.23±0.52 (0) | 0.177 | 0.57±0.97 (0) | 0.116 |
| pt297 | 22 | 0.95±4.26 (0) | 0.068 | 1.86±4.68 (0) | 0.061 | 2.82±8.65 (0) | 0.063 |
| pt298 | 38 | 3±1.51 (3) | -0.258 | 0.16±0.49 (0) | 0.232 | 3.16±1.67 (3) | -0.236 |
| pt299 | 52 | 4.79±4.16 (4) | 0.049 | 11.9±8.24 (11) | 0.166 | 16.69±11.33 (17.5) | 0.142 |
| pt300 | 20 | 1.45±0.83 (1) | 0.29 | 1.55±1 (1) | 0.116 | 3±1.72 (2) | 0.171 |
| pt301 | 20 | 1.3±2.77 (0) | -0.019 | 1.2±2.75 (0) | 0.229 | 2.5±5.22 (0) | 0.052 |
| pt302 | 24 | 1.96±2.12 (2) | -0.141 | 2.75±4.46 (2) | -0.253 | 4.71±6.36 (4) | -0.232 |
| pt303 | 24 | 1.42±1.38 (1) | -0.384 | 0.5±0.83 (0) | -0.518 | 1.92±1.77 (1.5) | -0.498 |
| pt304 | 33 | 1.16±1.44 (1) | -0.211 | 1.69±2.07 (1) | -0.098 | 2.84±3.36 (1.5) | -0.149 |
| pt305 | 24 | 0.17±0.56 (0) | 0.174 | 5.67±3.4 (6) | 0.385 | 5.83±3.51 (6) | 0.375 |
| pt306 | 24 | 0±0 (0) | NA | 0.17±0.82 (0) | 0.196 | 0.17±0.82 (0) | 0.196 |
| pt307 | 28 | 3.52±2.76 (4) | -0.319 | 1.96±2.14 (1) | -0.329 | 5.48±4.25 (5) | -0.32 |
| pt308 | 26 | 6.76±4 (5) | -0.045 | 2±1.87 (1) | -0.043 | 8.76±5.41 (7) | -0.042 |
| pt309 | 29 | 2.79±3.98 (1) | -0.377 | 5.97±6.67 (4) | 0.019 | 8.76±7.54 (7) | -0.073 |
| pt310 | 50 | 3.96±2.29 (4) | -0.154 | 1.94±2.04 (2) | -0.095 | 5.9±3.98 (6.5) | -0.146 |
| pt311 | 29 | 7.55±5.66 (7) | 0.039 | 8.62±8.13 (3) | 0.179 | 16.17±11.82 (10) | 0.141 |
| pt312 | 57 | 5.77±3.99 (5) | 0.073 | 7.95±5.04 (8) | 0.006 | 13.71±8.18 (12) | 0.017 |
| pt313 | 25 | 0±0 (0) | NA | 0.08±0.28 (0) | 0.112 | 0.08±0.28 (0) | 0.112 |
| pt314 | 27 | 0.67±1.07 (0) | -0.082 | 3±3.66 (2) | 0.222 | 3.67±3.71 (2) | 0.117 |
| pt315 | 26 | 11.19±3.27 (12) | -0.057 | 1.69±2.88 (0.5) | -0.181 | 12.88±5.08 (13) | -0.081 |
| pt316 | 41 | 1.9±3.32 (0) | -0.011 | 0.54±1.25 (0) | -0.233 | 2.44±3.69 (0) | -0.03 |
| pt317 | 31 | 0.82±1.25 (0) | -0.342 | 0.61±0.96 (0) | 0.009 | 1.43±1.93 (1) | -0.208 |
| pt318 | 31 | 1.55±1.48 (1) | 0.365 | 2.32±3.16 (1) | 0.473 | 3.87±4.4 (2) | 0.453 |
| pt319 | 25 | 5.19±5.79 (2) | 0.085 | 4.81±4.21 (4) | 0.286 | 10±9.08 (6) | 0.111 |
| pt320 | 28 | 0.64±0.49 (1) | -0.138 | 1±0.82 (1) | -0.094 | 1.64±0.99 (2) | -0.127 |
| pt321 | 78 | 11.04±5.16 (12) | 0.097 | 13.56±7.88 (14) | 0.07 | 24.6±11.54 (26) | 0.113 |
| pt322 | 26 | 0.46±2.35 (0) | -0.147 | 0.27±1.37 (0) | -0.147 | 0.73±3.73 (0) | -0.147 |
| pt323 | 23 | 0.13±0.34 (0) | -0.467 | 0.3±0.56 (0) | 0.099 | 0.43±0.73 (0) | -0.062 |
| pt324 | 26 | 4.38±3.18 (3) | 0.291 | 5.31±5.08 (4) | 0.093 | 9.69±7.79 (6.5) | 0.162 |
| pt325 | 28 | 3.19±1.73 (3) | -0.065 | 0.41±0.93 (0) | -0.041 | 3.59±1.91 (3) | -0.037 |
| pt326 | 26 | 3.56±4.59 (3) | -0.022 | 0.16±0.47 (0) | -0.229 | 3.72±4.59 (3) | -0.035 |

*Number of evaluations

**Mean±SD(median)

***Correlation between air pressure and each element of synovitis in each patient
